# Epidemiology of Hepatocellular Carcinoma in Taiwan

**DOI:** 10.3390/clinpract14020044

**Published:** 2024-03-28

**Authors:** Yu-Wei Lai, Ching-Hu Chung

**Affiliations:** 1 General Education Center, University of Taipei, Taipei 104, Taiwan; dai77@tpech.gov.tw; 2 Division of Urology, Taipei City Hospital Renai Branch, Taipei 106, Taiwan; 3 Department of Medicine, Mackay Medical College, New Taipei City 252, Taiwan

**Keywords:** hepatocellular carcinoma, incidence, comorbidities

## Abstract

Background: Hepatocellular carcinoma (HCC) is a major contributor to the world’s cancer burden. Understanding the HCC incidence rate in Taiwan is thus an interesting avenue of research. Methods: From an NHI database, those patients who had been newly diagnosed with HCC and who had been listed on a registry in a catastrophic illness dataset during the years 2013–2021 were enrolled in this study. Antineoplastic agent usage and comorbidities were also studied. Results: The incidence rate of HCC decreased from 57.77 to 44.95 in 100,000 from 2013 to 2021. The average age of patients with HCC increased from 65.54 years old with a CCI score of 4.98 in 2013 to 67.92 years old with a CCI score of 5.49 in 2021. Among these HCC patients, the patients under antineoplastic agent treatment decreased from 53.47% to 31.41% from 2013 to 2021. The presence of comorbidities in HCC patients was about 55.77–83.01% with mild liver disease and 29.93–37.30% with diabetes (without complications) in the period 2013–2021. Conclusions: The incidence rate of HCC slightly decreased in Taiwan. Due to antineoplastic agent usage decreasing over time, these results may indicate that more early-stage HCC patients detected in recent years were mainly treated with surgeries.

## 1. Introduction

Hepatocellular carcinoma (HCC) is the most common type of primary liver cancer and constitutes a significant burden for global health. It accounts for approximately 80% of all primary liver cancer cases worldwide, making it a major public health concern [1]. The incidence of hepatocellular carcinoma varies geographically, with the highest rates observed in Asia and sub-Saharan Africa. This variation can be attributed to regional differences in the prevalence of risk factors such as chronic viral hepatitis infections, particularly hepatitis B (HBV) and hepatitis C (HCV) viruses [2]. Other important risk factors include alcohol abuse, metabolic syndrome, aflatoxin exposure, and hereditary conditions such as hereditary hemochromatosis and alpha-1 antitrypsin deficiency [2,3]. Patients underling with nonalcoholic fatty liver disease continued to increase and also caused a significant portion of those with hepatocellular carcinoma during the current metabolic syndrome epidemic. HCC is a heterogeneous/complex disease, and multidisciplinary approaches are necessary in order to optimize its management. In contrast to other cancers, there are regional differences in etiology-dependent tumor biology [4,5].

HCC has a multifactorial etiology, with an interplay of genetic, epigenetic, and environmental factors contributing to its development. Genetic alterations, such as mutations in tumor suppressor genes (e.g., TP53 and PTEN) and the activation of oncogenes (e.g., CTNNB1 and MYC), drive the pathological transformation of hepatocytes into cancer cells (Nault and Zucman-Rossi, 2019). Epigenetic changes, including DNA methylation and histone modifications, further dysregulate gene expression, promoting tumor growth and survival. Environmental factors, such as chronic inflammation and oxidative stress, also play a critical role in hepatocarcinogenesis [6].

The clinical presentations of HCC can be diverse and nonspecific, leading to challenges in early diagnosis. In the early stages, patients may be asymptomatic or experience vague symptoms such as fatigue, abdominal discomfort, or weight loss. As the tumor progresses, more specific signs may manifest, including jaundice, hepatomegaly (enlarged liver), ascites (fluid accumulation in the abdomen), and liver dysfunction [7]. Diagnosis often involves a combination of imaging studies, such as ultrasound, computed tomography, and magnetic resonance imaging, along with serum biomarker analysis, including alpha-fetoprotein and des-gamma-carboxy prothrombin [8]. Tissue biopsy remains the gold standard for definitive diagnosis and determining tumor stage. The management of hepatocellular carcinoma depends on several factors, including tumor size, location, and extent of liver disease. Treatment options may include surgical resection, liver transplantation, locoregional therapies (such as radiofrequency ablation, transarterial chemoembolization, and radioembolization), systemic therapy (including targeted therapies and immune checkpoint inhibitors), and supportive care [2]. However, the prognosis for HCC remains poor, particularly in the advanced stages, due to the high rates of recurrence and limited treatment options.

Although the incidence rates of HCC have increased in many countries, in some countries, such as the US, these rates have abated in recent years. Understanding the HCC incidence rate in Taiwan is thus an interesting avenue of research. This retrospective cohort study aimed to identify newly diagnosed HCC patients in Taiwan and understand their demographic data, comorbidities, and antineoplastic agent usage.

## 2. Materials and Methods

### 2.1. Data Source

**Figure 1:**
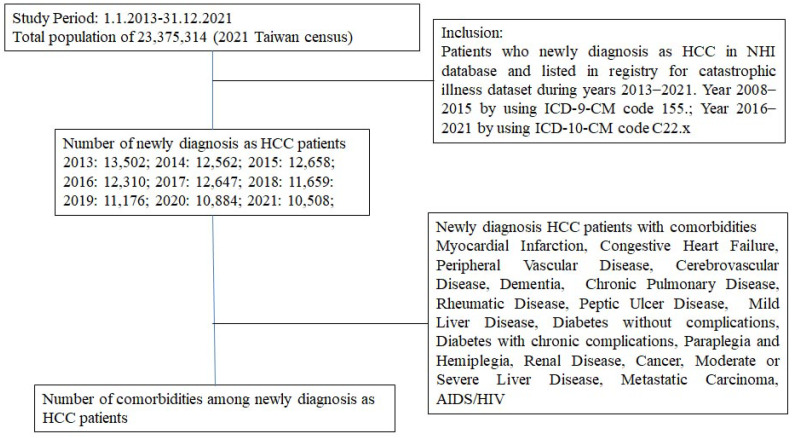
HCC patient enrollment flow.

The Taiwan NHIRD included 23 million beneficiaries in Taiwan. We used this database to establish a longitudinal medical history for each beneficiary by linking several computerized claim datasets. Information on newly diagnosed HCC patients was identified in the registry for catastrophic illness dataset during the years 2013–2021 and linked with the National Health Insurance Database through the civil identification number unique to each beneficiary. The study protocol was approved by the MacKay Memorial Hospital Institutional Review Board Taiwan, (Protocol Number: 20MMHIS230e, approval date: 10 August 2020). The NHIRD of Taiwan is a public database available through a formal application approved by the Health and Welfare Data Science Center of the Ministry of Health and Welfare, Taiwan (https://dep.mohw.gov.tw/DOS/np-2500-113.html, accessed on 15 January 2024). The enrollment flow is shown in Figure 1.

### 2.2. Study Inclusion

We examined the source population between 2013 and 2021. Patients were classified as newly diagnosed HCC patients according to the NHI database and if they were listed in the registry for catastrophic illness dataset during the years 2013–2021: ICD-9 cm code 155.x (malignant neoplasm of liver and intrahepatic bile ducts); ICD-10 cm code C22.x (malignant neoplasm of liver and intrahepatic bile ducts). The Charlson Comorbidity Index was used to identify comorbidities in HCC patients within one year before they were first listed in the registry for catastrophic illness dataset. We adopted Quan et al.’s coding [9]. Seventeen baseline covariates were selected, namely myocardial infarction, congestive heart failure, peripheral vascular disease, cerebrovascular disease, dementia, chronic pulmonary disease, rheumatic disease, peptic ulcer disease, mild liver disease, diabetes without complications, diabetes with chronic complications, paraplegia and hemiplegia, renal disease, cancer, moderate or severe liver disease, metastatic carcinoma, and AIDS/HIV. The detailed coding used in this study is shown in Supplementary Table S1. Antineoplastic agent usage was identified in newly diagnosed HCC patients who were prescribed this kind of treatment (ATC code: L01) in the index year.

### 2.3. Data Analyses

SAS 9.4 (SAS Institute Inc., Cary, NC, USA) was used for data analyses. Variable measures were identified based on the criteria described above. Frequencies or percentages were used to describe the categorical variables. To examine the linear gradient relationship with the risk of endpoints of interest, trend tests were also performed.

## 3. Results

### 3.1. Annual Incidence of HCC

**Figure 2:**
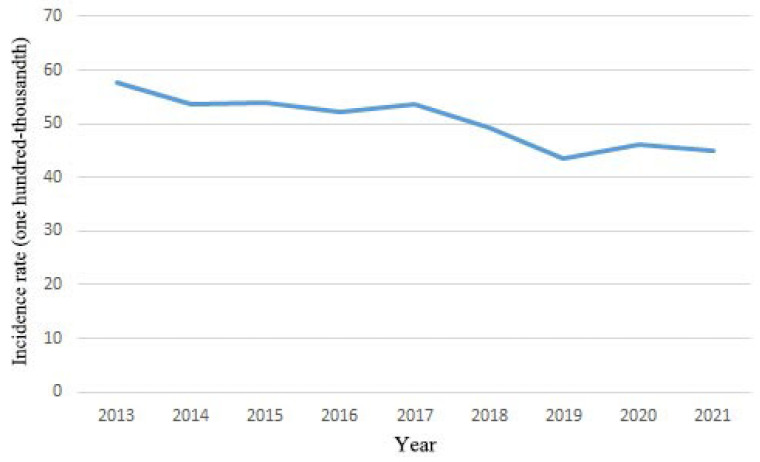
Annual incidence of HCC in the period 2013–2021. Patients were classified as newly diagnosed HCC patients if they were newly diagnosed with HCC in NHI database and listed in registry for catastrophic illness dataset during years 2013–2021: ICD-9 cm code 155.x (malignant neoplasm of liver and intrahepatic bile ducts); ICD-10 cm code C22.x (malignant neoplasm of liver and intrahepatic bile ducts).

Figure 1 shows the annual distribution of newly diagnosed HCC patient numbers between 2013 and 2021. The average annual incidence of HCC in Taiwan decreased from 57.77 in 100,000 in 2013 to 44.95 in 100,000 in 2021 (Figure 2; 57.77 in 2013, 53.61 in 2014, 53.88 in 2015, 52.29 in 2016, 53.65 in 2017, 49.43 in 208, 43.63 in 2019, 46.19 in 2020, and 44.95 in 2021, respectively) (*p* for trend <0.001).

### 3.2. Baseline Characteristics and Antineoplastic Agent Usage

**Figure 3:**
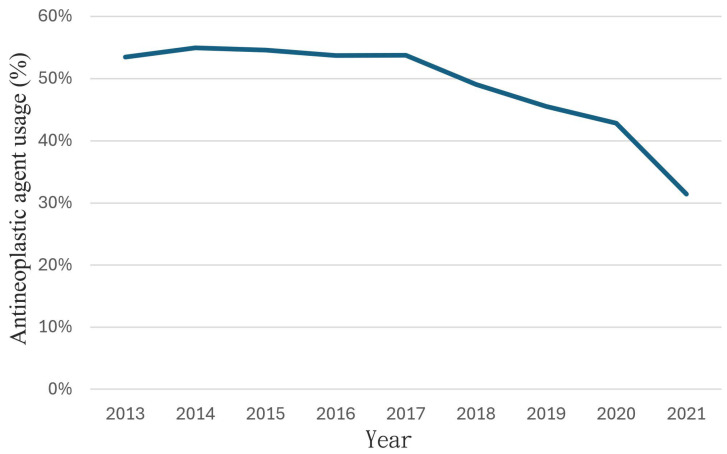
Antineoplastic agent usage in newly diagnosed HCC patients in the period 2013–2021. Antineoplastic agent usage was calculated as patients classified as newly diagnosed HCC patients prescribed antineoplastic agents (ATC code: L01) in index year.

We further identified the age and CCI among these newly diagnosed HCC patients during the years 2013–2021. The mean age at first HCC diagnosis increased from 65.54 years old to 67.92 years old in 2021 (Table 1; 65.54 in 2013, 65.88 in 2014, 66.51 in 2015, 66.68 in 2016, 66.84 in 2017, 67.41 in 208, 67.11 in 2019, 67.91 in 2020, and 67.92 in 2021, respectively) (*p* for trend <0.001). The CCI score at first HCC diagnosis also increased from 4.98 in 2013 to 5.49 in 2021 (Figure 1; 4.98 in 2013, 5.42 in 2014, 5.47 in 2015, 5.32 in 2016, 5.25 in 2017, 5.38 in 208, 5.35 in 2019, 5.51 in 2020, and 5.49 in 2021, respectively) (*p* for trend <0.001). Figure 3 shows the antineoplastic agent usage in newly diagnosed HCC patient numbers between 2013 and 2021. Antineoplastic agent usage in Taiwan decreased from 53.47% in 2013 to 31.41% in 2021 (Figure 3; 53.47% in 2013, 54.95% in 2014, 54.57% in 2015, 53.70% in 2016, 53.74% in 2017, 49.06% in 2018, 45.53% in 2019, 42.83% in 2020, and 31.41% in 2021, respectively) (*p* for trend <0.001).

### 3.3. Underlying Diseases in Newly Diagnosed HCC Patients

We further investigated the common underlying diseases in newly diagnosed HCC patients. The percentages of myocardial infarction, congestive heart failure, peripheral vascular disease, cerebrovascular disease, dementia, chronic pulmonary disease, rheumatic disease, peptic ulcer disease, mild liver disease, diabetes without complications, diabetes with chronic complications, paraplegia and hemiplegia, renal disease, cancer, moderate or severe liver disease, metastatic carcinoma, and AIDS/HIV within one year before HCC patients were first listed in the registry for catastrophic illness dataset were evaluated. The underlying diseases in newly diagnosed HCC patients generally increased regarding most comorbidities, but incidences of rheumatic disease, peptic ulcer disease, mild liver disease, and moderate or severe liver disease decreased over time (Table 2). The rates of cerebrovascular disease, chronic pulmonary disease, peptic ulcer disease, mild liver disease, diabetes without complications, diabetes with chronic complications, renal disease, cancer, moderate or severe liver disease, and metastatic carcinoma comorbidities were higher than 10%.

## 4. Discussion

This is a nationwide population-based study, based on the NHIRD in Taiwan, that systematically analyzed the epidemiology of HCC and its underlying diseases from 2013 to 2021. We determined that the number of newly diagnosed HCC patients decreased from 57.77 in 100,000 in 2013 to 44.95 in 100,000 in 2021 (Figure 2); these results are similar to those from other countries, such as the US [10]. Among these HCC patients, the average age increased from 65.54 years old with a CCI score of 4.98 in 2013 to 67.92 years old with a CCI score of 5.49 in 2021 (Table 1). Antineoplastic agent usage in these HCC patients decreased from 53.47% in 2013 to 31.41% in 2021 (Figure 3). The underlying diseases in newly diagnosed HCC patients decreased over time in terms of rheumatic disease, peptic ulcer disease, mild liver disease, and moderate or severe liver disease (Table 2). The rates of cerebrovascular disease, chronic pulmonary disease, peptic ulcer disease, mild liver disease, diabetes without complications, diabetes with chronic complications, renal disease, cancer, moderate or severe liver disease, and metastatic carcinoma comorbidities were higher than 10%.

The European Association for the Study of the Liver (ECSL) and the American Association for the Study of Liver Diseases (AASLD) recommend an abdominal ultrasound in patients with chronic liver disease or cirrhosis (high-risk HCC patients, every 6 months) for early-stage HCC screening [11,12]. However, the overall sensitivity for HCC screening is only 46%; for smaller lesions (less than 2 cm), this is only 21% and is substantially lower for lesions less than 1 cm [13]. The sensitivities of the Barcelona Clinic Liver Cancer staging system (BCLC) and the Milan standard for early-stage HCC screening are only 47% and 27.3%, respectively [14,15]. A deeper understanding of the molecular markers and underlying diseases, in conjunction with traditional pathology, may further improve the accuracy of HCC diagnosis. Few data sources, such as the claim database, are available for evaluating the underlying diseases among HCC patients. HCC is the most common type of primary liver cancer and is often associated with chronic liver diseases such as hepatitis B and hepatitis C infections, alcoholic liver disease, and nonalcoholic fatty liver disease or special populations with HCC, such as patients with obesity and elderly patients [16,17,18,19]. While chronic infections with hepatitis B virus, hepatitis C virus, and type 2 diabetes are widely recognized as comorbidities, numerous other conditions may remain undiagnosed [10,16]. Among the infectious factors of HCC, HBV infection is one of the most serious factors (especially in Asia). There are several mechanisms, including viral DNA integration, epigenetic changes, immune dysfunction, and viral gene mutations, which may cause HBV-related HCC [20]. Due to the World Health Organization’s encouragement of universal hepatitis B virus vaccination, the HBV vaccination in Taiwan has improved and has led to the prevention of mother-to-child transmission and the implementation of care to avoid the development of hepatocellular carcinoma [20]. HCV infection is one of the most serious factors in HCC. The DAA agents strategically intervene at various stages of the HCV replication life cycle, demonstrating remarkable efficacy with a brief treatment duration. Comprehensive studies consistently indicate that DAA therapy achieves a cure rate exceeding 90%, boasting a favorable safety profile and potentially yielding unforeseen advantages for patients [21,22]. The number of HCV patients was dramatically decreasing after the Ministry of Health and Welfare removed treatment restrictions [23].

Several studies have suggested a potential association between HCC and an increased risk of developing cardiovascular disease (CVD). Liver dysfunction can cause alterations in lipid metabolism, leading to increased levels of triglycerides and LDL cholesterol while decreasing HDL cholesterol levels. This dyslipidemia, combined with insulin resistance and obesity, can contribute to the development of cardiovascular risk factors such as hypertension, diabetes, and metabolic syndrome [24,25]. Furthermore, chronic inflammation plays a crucial role in both HCC and CVD. Similarly, chronic inflammation is a key contributor to the development and progression of atherosclerosis and CVD [26]. Additionally, shared risk factors, such as smoking, alcohol consumption, and obesity, contribute to both HCC and CVD. Tobacco smoking and heavy alcohol consumption have been implicated in the development of both diseases. Smoking can increase the risk of developing liver cancer in individuals with chronic hepatitis B or C virus infection [27]. Similarly, excessive alcohol consumption is a risk factor for both liver disease progression and the development of CVD [28]. It is worth mentioning that while there is growing evidence suggesting a correlation between HCC and CVD, further research is needed to understand the underlying mechanisms and establish a definitive causal relationship.

The correlation between hepatocellular carcinoma (HCC) and diabetes is an area of interest in medical research. Diabetes, particularly type 2 diabetes, has been identified as a potential risk factor for the development of HCC. Insulin resistance, hyperinsulinemia, and type 2 diabetes are characterized by insulin resistance, a condition in which the body’s cells do not respond effectively to insulin. This leads to compensatory hyperinsulinemia, which is an increased production of insulin by the pancreas. Insulin and insulin-like growth factor (IGF) signaling pathways play a role in cell growth, proliferation, and survival. Chronically elevated levels of insulin and IGFs may contribute to increased cell division and tumor growth in the liver, increasing the risk of HCC development [29].

Due to the decrease in antineoplastic agent usage, these results may indicate that there were more early-stage HCC patients detected in recent years that were mainly treated with surgeries (Figure 3). Several treatment modalities are used in the management of HCC, either alone or in combination. Surgical resection or liver transplantation may be considered for early-stage HCC when the tumor is confined to the liver and the patient is otherwise healthy. Liver transplantation offers the advantage of removing the tumor along with the underlying liver disease, reducing the risk of recurrence [30]. Locoregional therapies have been improved for patient selection and developed for a personalized approach to improve outcomes and reduce toxicity. These include procedures such as radiofrequency ablation, microwave ablation, transarterial chemoembolization, transarterial radioembolization, and percutaneous ethanol injection. Radiotherapy can be taken as an alternative way for surgical resection or locoregional therapeutic modalities or as a palliative modality [31]. For advanced-stage HCC, or when other treatments are not feasible, systemic therapy options, such as molecularly targeted therapy and immunotherapy, are used. Sorafenib, lenvatinib, regorafenib, and immune checkpoint inhibitors like nivolumab and pembrolizumab have shown efficacy in these settings [32]. Most guidelines commonly recommend immunotherapy (immune checkpoint inhibitor therapy) (atezolizumab plus bevacizumab), sorafenib, and lenvatinib as first-line options. As second-line options, regorafenib, cabozantinib, ramucirumab, nivolumab (with or without ipilimumab), and pembrolizumab have been recommended.

Although NHIRD data analysis provided benefits, they still had some limitations due to the nature of the NHIRD setup. First, self-payment medications, laboratory data, and height/weight (patient information) were lacking because of the objectives of the NHIRD. Second, disease severity is also unable to be measured. Third, NHIRD data analysis mainly relies on the international classification of diseases coding system since coding errors and hospital/physician differences can impact these results. Fourth, due to the healthcare system differences, demographics, and lifestyle factors, our results are universally applicable to other regions or countries and may contain some systematic errors. Finally, the time lag for the National Health Insurance Administration to release annual data is 18 months, and the most recent patients were unable to enroll in the study.

## 5. Conclusions

In conclusion, the incidence rate of HCC slightly decreased in Taiwan. Due to antineoplastic agent usage decreasing over time, these results may indicate that there were more early-stage HCC patients detected in recent years that were mainly treated with surgeries. Hepatocellular carcinoma is a significant global health issue, commonly arising in the context of chronic liver diseases such as viral hepatitis and cirrhosis. Its multifactorial etiology, diverse clinical presentation, and limited treatment options make it a formidable challenge for patients, healthcare providers, and researchers alike. Increased awareness, early detection, and the development of effective therapies are essential in combating this lethal disease and improving patient outcomes.

## Figures and Tables

**Table 1:** Age and CCI score among newly diagnosed HCC patients.

	Age			CCI		
Year	Mean	Median	95% CI	Mean	Median	95% CI
2013	65.54 ± 12.59	66	65.33–65.76	4.98 ± 2.62	18	4.94–5.03
2014	65.88 ± 12.63	66	65.66–66.10	5.42 ± 2.78	21	5.37–5.47
2015	66.51 ± 12.56	66	66.29–66.73	5.47 ± 2.78	19	5.42–5.52
2016	66.68 ± 12.45	67	66.46–66.90	5.32 ± 2.83	19	5.27–5.37
2017	66.84 ± 12.27	67	66.62–67.05	5.25 ± 2.82	18	5.20–5.30
2018	67.41 ± 12.15	67	67.19–67.93	5.38 ± 2.89	19	5.33–5.43
2019	67.11 ± 12.25	67	66.88–67.34	5.35 ± 2.87	18	5.30–5.40
2020	67.9112.24	68	67.68–68.14	5.51 ± 2.96	18	5.45–5.56
2021	67.92 ± 12.06	68	67.68–68.15	5.49 ± 2.95	19	5.43–5.55
*p* for trend	<0.001			<0.001		

**Table 2:** Underlying diseases in newly diagnosed HCC patients.

	2013	2014	2015	2016	2017	2018	2019	2020	2021	*p* for Trend
Myocardial Infarction	0.84%	1.13%	1.02%	1.18%	1.27%	1.56%	1.44%	1.52%	1.65%	<0.001
Congestive Heart Failure	5.47%	6.33%	6.54%	6.83%	7.29%	7.21%	7.02%	8.16%	7.14%	<0.001
Peripheral Vascular Disease	2.18%	2.71%	2.88%	2.83%	2.59%	2.49%	3.02%	2.78%	2.81%	<0.001
Cerebrovascular Disease	8.10%	10.36%	10.43%	10.08%	10.39%	10.78%	11.02%	10.63%	10.57%	<0.001
Dementia	2.51%	3.57%	3.79%	3.95%	4.13%	4.82%	4.86%	5.15%	4.78%	<0.001
Chronic Pulmonary Disease	11.49%	15.70%	15.44%	15.74%	15.70%	15.84%	15.43%	14.70%	13.27%	<0.001
Rheumatic Disease	1.73%	1.93%	2.08%	1.84%	1.29%	1.45%	1.39%	1.30%	1.28%	<0.001
Peptic Ulcer Disease	28.77%	33.64%	33.48%	33.02%	31.34%	30.87%	29.45%	29.45%	27.55%	<0.001
Mild Liver Disease	79.61%	83.01%	82.52%	65.35%	56.81%	57.53%	57.97%	56.63%	55.77%	<0.001
Diabetes without complications	29.93%	33.53%	34.22%	34.72%	35.27%	35.98%	35.92%	36.51%	37.30%	<0.001
Diabetes with chronic complications	8.21%	10.10%	11.22%	12.23%	13.32%	14.62%	14.92%	15.99%	15.59%	<0.001
Paraplegia and Hemiplegia	0.64%	0.85%	0.92%	0.74%	0.66%	0.74%	0.90%	0.94%	0.91%	<0.001
Renal Disease	9.35%	11.20%	12.56%	13.30%	13.15%	14.47%	14.32%	15.11%	15.31%	<0.001
Cancer	96.49%	96.68%	96.73%	96.49%	96.77%	96.71%	96.28%	96.75%	96.56%	<0.001
Moderate or Severe Liver Disease	10.53%	12.17%	12.03%	10.33%	8.90%	9.35%	8.32%	8.86%	8.06%	<0.001
Metastatic Carcinoma	11.06%	12.54%	12.47%	13.26%	13.91%	14.49%	14.81%	16.47%	17.43%	<0.001
AIDS/HIV	0.09%	0.14%	0.10%	0.07%	0.11%	0.21%	0.13%	0.17%	0.22%	<0.001

## Data Availability

This study is based on data from Taiwan’s Health and Welfare Data Science Centre at the Ministry of Health and Welfare (H109213). The data underlying this study belong to the National Health Insurance Research Database (NHIRD) of Taiwan and cannot be made publicly available due to legal restrictions. However, the data are available through a formal application to the Health and Welfare Data Science Centre at the Ministry of Health and Welfare, Taiwan (https://dep.mohw.gov.tw/DOS/np-2500-113.html, accessed on 15 January 2024) and require a signed affirmation regarding data confidentiality. The authors have no special privileged access to the database.

## Supplementary Materials

The following supporting information can be downloaded at https://www.mdpi.com/article/10.3390/clinpract14020044/s1, Table S1: Coding detail.
