# Supplementary material for: Epidemiology of Hepatocellular Carcinoma in Taiwan
Source: Clin Pract. 2024 Mar 28;14(2):570–8. doi: 10.3390/clinpract14020044 (PMC11048999; doi:10.3390/clinpract14020044)
Supplement: Supplementary file 1 [file clinpract-14-00044-s001.zip › clinpract-2859880-supplementary.pdf]

Table S1. Coding detail.

Comorbidity	ICD-9	ICD10
Myocardial Infarction	410.x, 412.x	I21.x, I22.x, I25.2
Congestive Heart Failure	398.91, 402.01, 402.11, 402.91, 404.01, 404.03, 404.11, 404.13, 404.91, 404.93, 425.4-425.9, 428.x	I09.9, I11.0, I13.0, I13.2, I25.5, I42.0, I42.5-I42.9, I43.x, I50.x, P29.0
Peripheral Vascular Disease	093.0, 437.3, 440.x, 441.x, 443.1-443.9, 47.1, 557.1, 557.9, V43.4	I70.x, I71.x, I73.1, I73.8, I73.9, I771, I79.0, I79.2, K55.1, K55.8, K55.9, Z95.8, Z95.9
Cerebrovascular Disease	362.34, 430.x-438.x	G45.x, G46.x, H34.0, T60.x-I69.x
Dementia	290.x, 294.1, 331.2	F00.x-F03.x, F05.1, G30.x, G31.1
Chronic Pulmonary Disease	416.8, 416.9, 490.x-505.x, 506.4, 508.1, 508.8	I27.8, I27.9, J40.x-J47.x, J60.x-J67.x, J68.4, J70.1, J70.3
Rheumatic Disease	446.5, 710.0-710.4, 714.0-714.2, 714.8, 725.x	M05.x, M06.x, M31.5, M32.x-M34.x, M35.a, M35.3, M36.0
Peptic Ulcer Disease	531.x-534.x	K25.x-K28.x
Mild Liver Disease	070.22, 070.23, 070.32, 070.33, 070.44, 070.54, 070.6, 070.9, 570.x, 571.x, 573.3, 573.4, 573.8, 573.9, V42.7	B18.x, K70.0-K70.3, K70.9, K71.3-K71.5, K71.7, K73.x, K74.x, K76.0, K76.2-K76.4, K76.8, K76.9, Z94.4
Diabetes without complications	250.0-250.3, 250.8, 250.9	E10.0, E10.1, E10.6, E10.8, E10.9, E11.0, E11.1, E11.6, E11.8, E11.9, E12.0, E12.1, E126, E12.8, E12.9, E14.0, E14.1, E14.6, E14.8, E14.9
Diabetes with chronic complications	250.4-250.7	E10.2-E10.5, E10.7, E11.2-E11.5, E11.7, E12.2-E12.5, E12.7, E13.2-E13.5, E13.7, E14.2-E14.5, E14.7
Paraplegia and Hemiplegia	334.1, 342.x, 343.x, 344.0-344.6, 344.9	G04.1, G11.4, G80.1, G80.2, G81.x, G82.x, G83.0–G83.4, G83.9
Renal Disease	403.01, 403.11, 403.91, 404.02, 404.03, 404.12, 404.13, 404.92, 404.93, 582.x, 583.0–583.7, 585.x, 586.x, 588.0, V42.0, V45.1, V56.x	I12.0, I13.1, N03.2–N03.7, N05.2–N05.7, N18.x, N19.x, N25.0, Z49.0–Z49.2, Z94.0, Z99.2
Cancer	140.x–172.x, 174.x–195.8, 200.x–208.x, 238.6	C00.x–C26.x, C30.x–C34.x, C37.x–C41.x, C43.x, C45.x–C58.x, C60.x–C76.x, C81.x–C85.x, C88.x, C90.x–C97.x
Moderate or Severe Liver Disease	456.0–456.2, 572.2–572.8	I85.0, I85.9, I86.4, I98.2, K70.4, K71.1, K72.1, K72.9, K76.5, K76.6, K76.7
Metastatic Carcinoma	196.x-199.x	C77.x-C80.x
AIDS/HIV	042.x-044.x	B20.x-B22.x, B24.x
